# Pharmacoepidemiology Research-Real-World Evidence for Decision Making

**DOI:** 10.3389/fphar.2021.723427

**Published:** 2021-09-07

**Authors:** Anick Bérard

**Affiliations:** ^1^ Faculty of Pharmacy, University of Montreal, Montreal, QC, Canada; ^2^ Faculty of Medicine, Université Claude Bernard Lyon 1, Lyon, France; ^3^ CHU Sainte-Justine, Montreal, QC, Canada

**Keywords:** pharmacoepidemiology, real-world evidence, real-world data, state-of-the-art research, decision making, data access, underrepresented populations

## Introduction

Pharmacoepidemiology is the study of the utilisation and effects of medications in large human populations, and it is a bridge science spanning both clinical pharmacology and epidemiology. Over the years, pharmacoepidemiology has benefited from its ability to synergize multiple disciplines including epidemiology, pharmacology, medicine, biostatistics, and social sciences. Real-World Evidence (RWE) in pharmacoepidemiology is the clinical findings on usage, benefits and risks of a medication generated from the analysis of real world data (RWD) (Leveraging Real World Evidence in Regulatory Submissions of Medical Devices, 2021). The real-life clinical impact of a medication might be more clearly demonstrated through RWD and RWE given that controlled clinical trials often cannot evaluate all applications of a drug in clinical practice across the full range of potential users. Indeed, clinical trials often have very strict inclusion and exclusion criteria, measure standardize outcomes, have short and strict follow-up rules, and can randomize medication exposures among study subjects, making them the design of choice to quantify the efficacy of a medication (Scandinavian Simvastatin Survival Study Group, 1994; Evans, 2010). Clinical trial efficacy results remain the main indicators on which decisions are usually made to determine whether drugs are available at bedside or as outpatient treatments. However, they do not directly predict clinical effectiveness since medications are often prescribed for unapproved conditions, with less than optimal patient observance to prescribed regimens; and they usually have follow-up periods that are often too short to address long-term effectiveness or safety (Scandinavian Simvastatin Survival Study Group, 1994). As such, many findings from clinical trials cannot be replicated in clinical practice, hence the need for pharmacoepidemiologic studies and RWE. For example, the effectiveness of statins to treat vascular diseases is much lower than the efficacy shown in clinical trials due in parts to the low observance and adherence to treatment regimens in the context of primary prevention (Heart Protection Study Collaborative Group, 2002; Topol, 2004; Goldenberg and Glueck, 2009). Furthermore, clinical trials are not the best design to assess the safety of a medication (adverse events especially when they are rare). Rofecoxib was taken off the market due to increased risk of cardiovascular events and stroke when used in real-life settings (outside of the authorized indications, or for longer time-periods, etc.) (Mukherjee et al., 2001; Singh and Loke, 2012). Since the Thalidomide disaster, pregnant women have been systematically excluded from clinical trials. However, the Tri-Council Policy Statement on the Ethical Conduct for Research Involving Humans (TCPS2 2018) states, under Article 4.3, “Women shall not be inappropriately excluded from research solely on the basis of their reproductive capacity, or because they are pregnant or breastfeeding” (Canadian Institutes of Health Research NSaERCoC, and Social Sciences and Humanities Research Council, 2005). Moreover, since 1994, the Institute of Medicine has recommended that pregnant women be presumed eligible for participation in clinical studies (Mastroianni et al., 1994). Despite this, the systematic exclusion of pregnant and breastfeeding persons remains a common practice. In 2011–2012, pregnant women were excluded from 95% of phase IV interventional industry-sponsored trials (Shields and Lyerly, 2013). Further, 98% of the 172 drugs approved in the United States between 2000 and 2010 had insufficient data to determine the risk of developmental toxicity in humans (Adam et al., 2011). Although clinical trial methodology would need to be adapted in pregnant studies, by potentially having an active comparator, efficacy results could have a clear impact on mothers’ and newborns’ overall health. Clinical trials also exclude persons with multicomorbidities even if they represent 52% of the overall population (Islam et al., 2014); trials also exclude persons with communication limitations or other limitations of various kinds, which limits equity and representation. Pharmacoepidemiologic studies on large observational longitudinal cohorts on the risks and potential benefits of medication use are the only way to bridge the knowledge gap, using and linking health/demographic/hospital data routinely collected, and already available to and accessible by researchers, RWD.

In recent years however, large national administrative databases or registries, big data, have been increasingly used in the field of pharmacoepidemiology, recognizing the importance of large size longitudinal population-based cohorts. Healthcare electronic records, registries, medical claims, pharmacy data, and data from wearable and mobile technology have also become a cornerstone in the process of assessing performance and providing feedback to improve quality of health care delivery and outcomes at a population-level (Klonoff, 2020). The integrated system of health care delivery in many countries has facilitated the linking of inpatient, outpatient [including emergency department (ED) visits], hospital, ambulatory care, pharmaceutical, immunization, and laboratory data. Indeed, the healthcare system results in the accumulation of a wealth of RWD data annually representing real-life use of services, diagnoses, hospitalisations and medication use that can be used for policymaking and guidelines to increase wellbeing and decrease risk. Digitization of health care provides new opportunities to close the gap between research and clinical care. Big data such as routinely collected health care data also gives the potential for the detection of infrequent events, as well as long-term infrequent outcomes. RWE using RWD also requires lower resource intensity and allows rapid answers to pressing questions. The next challenges are how best to use and analyse these data, and the importance of harmonizing worldwide cohorts in order to better compare inter-study findings and have public policy applications. Quality and valid RWD must use standardized and harmonized codes, algorithms, terminologies and vocabularies, especially when pooling data from different data sources (electronic health records, billing or hospitalisation databases, etc.). This is defined as the Common data models or formats (Reisinger et al., 2010; Schneeweiss et al., 2020). Having harmonized cohorts ready to be used will further expedite investigations, and allow for near real-time queries, and greater impact on patient care. Finally, RWE also has the potential to fill unmet needs in subpopulations routinely excluded from clinical trials such as pregnant women, children, and the very old with multicomorbidities as well as for sub-groups underrepresented in studies such as those with limitations, immigrants, racialized groups, and First Nations.

Today, large datasets and big-data analyses are crucial to advancing research and treatments. Hence, it is of utmost importance to have more representative and large cohorts with a longer follow-up to: 1) study real-world prescribing and use of new drugs that have been newly marketed; 2) study rare adverse events for which large sample sizes are required for the analyses; 3) study adverse outcomes or conditions that occur or are diagnosed later such as cancers or metabolic disorders; or 4) assess the impact of sociodemographic variables or insurance plan (access, restrictions, and reimbursement) on the occurrence of adverse health outcomes. RWE improves how research data is organized, accessed or used, which allows for fast-track research and relevance. This is even more important in times of crises such as in the COVID-19 pandemic. Furthermore, shortcomings from clinical trials can be addressed by linking clinical trial data with RWD, which can enhance evidence provided to decision makers.

The ultimate goal of cohorts put in place with the use of RWD is to identify serious events associated with medication use in a timely manner, which allows us to take preventive measures in order to avert or minimize them. In addition, there is a great need, and an added value for such RWE cohorts given country-specific cultural diversity, which is rarely explored in studies. These cohorts have value by identifying and quantifying differences in prescribing practices and medication use as well as identifying risk profiles of medication users.

## Fast Access to Real World Data to Address Ongoing Crises–Example of the COVID-19 Pandemic

The recent pandemic has highlighted the importance and the need of on-going preparedness in virology, vaccine technology, public health infrastructure, and rapid real-time access to valid data. Although one can argue that some systems were better prepared than others, we can all reflect and acknowledge the fact that no country had fast enough unconditional access to RWD to fully comprehend the extent of the pandemic in real-time as well as measure the impact of its public health measures/restrictions on the spread of COVID-19. This led to preventable mortality and adverse events and comorbidities that will likely be felt for years to come. At the time when already available medications were being tested as potential COVID-19 therapeutics in clinical trials, RWD should have been readily available and used to assess safety and effectiveness for the treatment of COVID-19. Although observational research is more prone to biases than randomized clinical trials (Pandis, 2014), RWE emerging from the use of these large real-life databases would have given immediate findings that could have been used to treat patients with COVID-19 during the very first wave of the pandemic. It would have been especially important when the decision was made in the US to use hydroxychloroquine as an emergency COVID-19 treatment based on theoretical effectiveness (Thomson and Nachlis, 2020). This led to increased demand and prescriptions for hydroxychloroquine to treat COVID-19 even when later efficacy data showed no effect, which resulted in shortage of the drug to treat indicated conditions such as rheumatoid arthritis or lupus.

Patient data confidentiality is often used as the main reason to delay access to real-world billing or hospital data. This is because there is a misunderstanding on what is provided to researchers. When patient consent is not possible, large RWD provided to researchers are anonymized, which makes identification of patients extremely unlikely. Although we can agree that privacy is important and necessary, RWD accessibility criteria cannot be too strict to discourage usage, especially when such data is the key to improved health, and treatments. Large RWD are the cornerstone for valid and rapid real-time measurement of effects in pharmacoepidemiology, especially in times of crisis. This needs to be understood and valued by governments, decision makers, and the lay public. Outreach from researchers and funding agencies to decision makers and the lay public is urgently needed.

## Real-World Evidence for Decision Making

**FIGURE 1:**
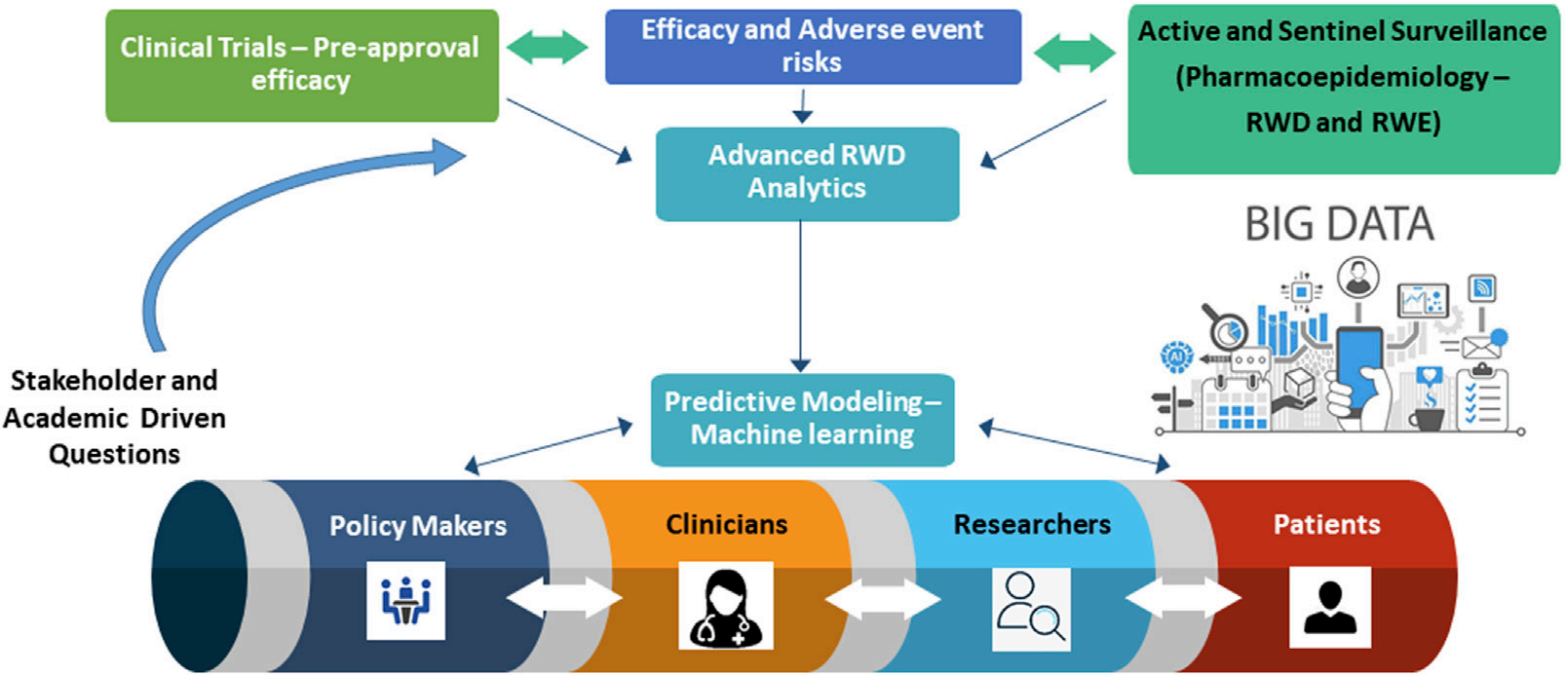
Pharmacoepidemiology Research Paradigm - from research to policy to bedside.

RWE can be leverage to bring new medications to market, evaluate the safety and effectiveness of existing medications for new uses, and assess the continued performance and safety of products once on the market, across the drug total life cycle (Leveraging Real World Evidence in Regulatory Submissions of Medical Devices, 2021). The FDA, Health Canada and the European Medicines Agency are starting to rely on RWE to support their regulatory decisions to help speed patient access to innovations that advance public health (Honig, 2021). It is however essential to distinguish between the sources of RWD and the evidence derived from that data. The quality of data and how they were collected as well as the validity of the methodology in the context of regulatory decision-making need to be assessed as decisions can have life-threatening impacts. Regulatory bodies have a long history of using RWE to monitor and evaluate the safety of drug products after they are approved (post-market). However, there is significant interest in and gradual acceptance of a potentially broader role for RWE in regulatory decision-making (Figure 1). For example, the FDA has accepted RWE to support drug product approvals, primarily in the setting of oncology and rare disease, areas in which clinical trials are challenging to do (Leveraging Real World Evidence in Regulatory Submissions of Medical Devices, 2021). When reviewing the use of RWE to support a regulatory decision, decision bodies have relied on scientifically robust methods and approaches to determine whether the submitted RWE is of sufficient quality to support the decision. Another challenge will be to improve RWE methodology and define the quality aspects of RWE studies to ensure that they possess the necessary reliability and gain broader acceptance among regulators and other stakeholders. Recently, the International Society for Pharmacoepidemiology has published guidelines for the planning and dissemination of pharmacoepidemiologic studies in an attempt to increase validity and standardization of reporting (Andrews et al., 1996).

Valid and reproducible RWE that will be used for decision making, and clinical practice guidelines based on state-of-the-art infrastructures are urgently needed as well as real-time rapid access to data. Pharmacoepidemiologic studies using RWD are needed to assess causality, understand discrepancies between populations, and quantify rare events; this is even truer in populations excluded from the majority of clinical trails.
